# Network approaches for modeling the effect of drugs and diseases

**DOI:** 10.1093/bib/bbac229

**Published:** 2022-06-16

**Authors:** T J Rintala, Arindam Ghosh, V Fortino

**Affiliations:** Institute of Biomedicine, University of Eastern Finland, 70210 Kuopio, Finland; Institute of Biomedicine, University of Eastern Finland, 70210 Kuopio, Finland; Institute of Biomedicine, University of Eastern Finland, 70210 Kuopio, Finland

**Keywords:** biological networks, network analysis, disease modeling, drug’s MOA

## Abstract

The network approach is quickly becoming a fundamental building block of computational methods aiming at elucidating the mechanism of action (MoA) and therapeutic effect of drugs. By modeling the effect of drugs and diseases on different biological networks, it is possible to better explain the interplay between disease perturbations and drug targets as well as how drug compounds induce favorable biological responses and/or adverse effects. Omics technologies have been extensively used to generate the data needed to study the mechanisms of action of drugs and diseases. These data are often exploited to define condition-specific networks and to study whether drugs can reverse disease perturbations. In this review, we describe network data mining algorithms that are commonly used to study drug’s MoA and to improve our understanding of the basis of chronic diseases. These methods can support fundamental stages of the drug development process, including the identification of putative drug targets, the *in silico* screening of drug compounds and drug combinations for the treatment of diseases. We also discuss recent studies using biological and omics-driven networks to search for possible repurposed FDA-approved drug treatments for SARS-CoV-2 infections (COVID-19).

## Introduction

Biological systems are highly complex involving many heterogeneous elements interacting with each other and forming subsystems at different levels of organization. Network medicine aims to address this complexity by considering the system holistically rather than focusing on only the set of direct disease genes or drug targets [1]. Molecular level interactions have been studied extensively in the context of disease mechanisms and drug discovery. As a result, information about different relations among and between drugs, diseases, and proteins as well as other related concepts is readily available in many databases. This can be naturally represented as networks where the vertices/nodes represent different molecules, and the edges/links represent relations or interactions. Network data mining (NDM) algorithms can be used to detect interesting structural properties of these networks which can be useful in identifying relevant mechanisms for disease subtyping, prognosis, and drug discovery. However, it is also possible to use NDM algorithms to predict novel associations between elements without explicitly considering the mechanisms. For example, by utilizing the connections and the guilt-by-association principle it is possible to uncover previously unknown relations (e.g. drug-target or disease-drug associations) based on how closely connected different entities are in the network. Other essential state-of-the-art methods are those based on matrix factorization and graph neural networks. Matrix factorization has been extensively used for drug repurposing based on the use of heterogeneous networks, while graph neural networks can combine the extraction of drug- and disease-relevant features with the prediction task at hand to achieve a higher accuracy. In this review, we will first describe various types of biological networks and basic NDM algorithms. Then, we describe and compare methods that utilize network-based approaches for the identification of drug-targets or drug-disease associations, and the prediction of drug combination or drug sensitivity. We also examine applications of these methods in COVID-19 drug repurposing.

## Construction of biological networks

**Figure 1:**
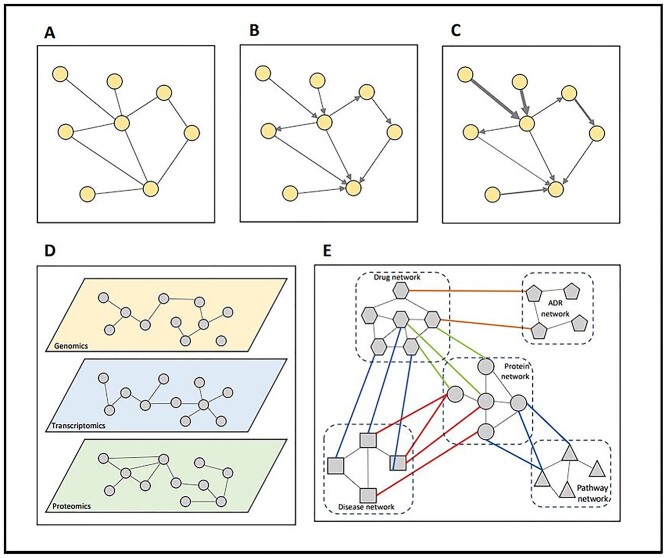
Different types of networks. (A) An undirected-unweighted network provide information only regarding the possible connections between the nodes. No information is provided regarding the type of interaction or its strength. (B) Directed network on the other hand provides information about the direction of interaction, (C) while weighted network tell about the strength of the interaction often denoted by edge. (D) Multiplex networks are formed by obtaining interaction information from different sources for the same set of nodes. Each layer on a multiplex network refers to interaction from different sources. As the nodes within each layer is of similar type, the network within each layer could be referred to as homogenous network. (E) In contrast to homogenous network, heterogenous network involves interaction between different types of nodes.

Biological networks, in general, can be categorized into different types based on how it has been constructed, the content of the network and the data that was used for the construction of the network. A network may be directed or undirected depending if the edges of the networks provide information on the direction of interaction between the nodes. Pathway-based networks are usually represented as directed graphs, while gene-gene co-expression networks on the other hand are usually undirected networks as though they talk about the strength of co-expression. Since such networks contain information regarding the strength of interaction, the networks are also referred to as weighted networks. Unweighted networks do not provide information regarding the strength of interaction. Further, depending on the type of nodes a network has, it may be classified as homogeneous and heterogeneous networks. Homogeneous networks contain only one type of nodes while heterogeneous networks may contain nodes of different types, e.g. drug–disease–gene network. Heterogeneous networks have shown to be promising in exploring the interplay of drugs and diseases by connecting available multifaceted data in a single framework. These networks can be constructed by either aggregating individual interaction information between different biological entities from literature (referred to as curated networks or knowledge-based networks) or from high-throughput data (referred as data-driven networks). Given the wide range of experiments currently available for studying interactions and for generation of high-throughput data, the information may be either aggregated to form a single network or a different network can be constructed based on different types of interactions (e.g. protein interactions or gene co-expression). While in the first approach the source of interaction is hidden, the second method does not allow to combine complementary knowledge derived from different biological relationships. Multiplex networks provide a possible solution to the problem by allowing integration of interaction information from different experimental types as different layers. However, there are issues that might arise when building and using different biological networks involving different biological entities. First, cross-layer relations are often not supported by experimental data. Besides, those that are inferred by computational methods could be potentially biased (e.g. experimental data are often available for commonly studied diseases such as cancer) [2]. Furthermore, the tissue or cell specificity is often hidden in heterogenous networks. Finally, basic NDM algorithms need to be re-designed for heterogenous and multiplex networks [3]. Figure 1 graphically illustrates different types of networks that can be built to study the mechanism of actions (MoA) of drugs and diseases.

### Knowledge-based networks

Knowledge-based networks are created by aggregating interaction information between different biological entities that are spread across the literature. The process of curation of knowledge-based networks might be tedious and time-consuming but is comparatively robust as it usually involves manual inspection of each interaction information in the published literature. Furthermore, knowledge-based networks might not be specific to a particular biological condition such as diseased states and thus might not be useful for inferring relations that are changed dynamically across conditions. Also, it has been observed that the amount of experimentally validated interactions available is usually skewed towards a few well-studied genes or diseases or other biological entities. Several databases are currently available that collect interaction and/or association events that can aid in understanding effects of drugs and diseases. But these databases are usually specific to only a type of interaction. Table 1 lists different types of interactions that can be retrieved from different databases. Two widely used interaction databases are BioGRID [4, 5] and StringDB [6], which have been extensively used to study the interactions between the mode of action of drugs and the molecular mechanisms dysregulated by diseases. Both databases aim to define different types of interaction. For instance, STRING contains interaction information obtained from five major sources including automated text mining of scientific literature, primary protein–protein interaction knowledgebases, co-expression, high-throughput experiments and genomic context prediction. PPI networks can also be built using annotation databases, such as the Kyoto Encyclopedia of Genes and Genomes (KEGG) [7] and REACTOME [8].

DrugBank is another widely used resource providing comprehensive information about approved as well as experimental and investigational drugs [9]. Besides molecular information of each drug, it also provides information pertaining to associations between drugs, drug-targets, and diseases. DrugBank is also one of the largest resources of manually curated drug–drug interactions reporting both adverse and beneficial interactions. Complementing the efforts of DrugBank are the ADReCs and SIDER databases that collect information about reported adverse drug reactions for marketed medicines and can form an interesting resource for understanding drug toxicity mechanisms [10, 11]. Further association between genes and diseases can also be retrieved from databases like DisGeNET [12], OpenTargets [13] and PharmGKB [14] to create a bipartite network between diseases and related genes.

In view of the recent COVID-19 pandemic, several of these databases have stepped up efforts to create a dedicated section containing information related to covid. For example, IntAct [15] has assembled about 10 000 protein–protein and RNA-protein interactions involving SARS-CoV and SARS-CoV2. Therapeutic Target Database (TTD) has created a comprehensive collection of anti-coronavirus drugs along with therapeutic targets. DrugBank’s COVID-19 section provides details on approved and unapproved drugs and potential targets.

**Table 1:** A list of databases to build knowledge-driven networks. The rows indicate the databases, while the columns indicate the types of connections that can be implemented

	Protein–Protein	Protein-Pathway	Gene-Variant	miRNA-Gene target	Drug-Target/ Gene-Chemical	Drug-Disease/ Chemical-Disease	Drug–Drug/ Chemical–Chemical	Drug-ADR	Chemical-Variant	Drug-Pathway/ Chemical-Pathway	Disease-Gene	Disease-miRNA	Disease-Disease	Disease-Pathway	Disease-Variant	ADR-Protein	ADR-Variant	References	Last updated
ADReCS								√										[106]	2021
ADReCS-Target								√								√	√	[10]	2017
Agile Protein Interactomes DataServer (APID)	√																	[107]	2021
BioGRID	√				√													[5]	2022
Comparative Toxicogenomics Database (CTD)		√			√	√					√			√				[108]	2022
DisGeNet											√		√		√			[109]	2020
DrugBank					√		√		√	√								[9]	2022
Human microRNA Disease Database (HMDD)												√						[110]	2019
Kyoto Encyclopedia of Genes and Genomes (KEGG)		√																[111]	2022
miRTarBase				√														[112]	2021
Online Mendelian Inheritance in Man (OMIM)											√							[113]	2022
OpenTarget											√							[13]	2022
PharmGKB			√		√		√		√						√			[14]	2022
REACTOME	√	√								√								[114]	2022
Small Molecule Pathway Database (SMPDB)		√								√								[115]	2013
STITCH					√		√											[116]	2015
STRING	√																	[6]	2021
Therapeutic Target Database (TTD)		√			√	√					√							[117]	2022

**Table 2:** A list of databases to build data-driven networks. The rows indicate the databases, while the columns indicate omics data types

	Chemical structure	Genomic	Transcriptomic	Proteomic	Metabolomic	Epigenomic	Drug sensitivity	References
Chemical Entities of Biological Interest (ChEBI)	√							[118]
Human Metabolome Database (HMDB)	√				√			[119]
The Cancer Genome Atlas Program (TCGA)		√	√	√		√		[120]
Genotype-Tissue Expression (GTEx)		√	√					[121]
Clinical Proteomic Tumor Analysis Consortium (CPTAC)				√				[122]
DrugMatrix (data available via GEO)			√					[123]
Gene Expression Omnibus (GEO)			√					[124]
Open TG-GATEs			√					[17]
Library of Integrated Network-Based Cellular Signatures (LINCS) (data available via GEO)			√					[125]
Genomics of Drug Sensitivity in Cancer							√	[126]
European Nucleotide Archive (ENA)		√	√			√		[127]
PRoteomics IDEntifications (PRIDE) Archive				√				[128]
Catalogue Of Somatic Mutations In Cancer (COSMIC)		√						[129]

### Data-driven networks

Data-driven networks can be built upon high-throughput experimental data (Table 2). These networks aim to indicate putative biomolecular interaction within a specific biological condition or disease, or even profiling patient-specific networks. For example, gene expression profiling using microarray and RNA sequencing has been extensively used to define co-expression networks that can aid in understanding the changes in interaction patterns that occur due to drug or chemical intervention [16, 17] or between normal and diseased states [18]. However, a major issue with such data-driven networks is that it can be noisy, and usually a large number of samples are required for building robust networks. Other forms of omics data like genomic, epigenomic, metabolomic and proteomic have also gained importance for understanding the mechanism of diseases and drug action, and accordingly network-based approaches have also been explored. For example, using methylation profiles to create weighted co-methylation networks, Xu *et al.* [19] was able to identify eight methylated CpG islands that could distinguish HIV/TB co-infected patients from HIV mono-infected patients. Each of these different types of omics data can be used individually, but the growth of large-scale multi-omics data projects like the Cancer Cell Line Encyclopedia (CCLE) Project [20], Genomics of Drug Sensitivity in Cancer (GDSC) [21], NCI-60 pan cancer data and The Cancer Genome Atlas (TCGA), which provides reliable data to construct robust network, has also aided in the extensive use of multi-omics networks. CCLE, GDSC and NCI-60 pan cancer projects have been widely used to decipher the underlying molecular mechanisms of cancer in response to drug using network biology approaches. While TCGA includes large-scale genomics profiles derived from tumor tissues, CCLE, GDSC and NCI-60 contain anticancer drug response data and omics profiles of cancer cell lines. Networks created from such multi-perspective data can help to elucidate disease networks in a more holistic way and predict therapeutics [22]. However, integration of multi-omics data is often not straightforward due to the differences in measured entity and their probabilistic distribution in each omics data type. As such, different methods have been explored and can be grouped into two types—vertical where multiple omics data from the same set of samples are integrated and horizontal where data from different biological samples are mapped to shared or related entities [23]. At the simplest level, sample-sample similarity networks can be created based on different types of multi-omics data followed by fusing them into one network to represent the full spectrum of underlying data [24]. Another approach based on horizontal integration of multiomics data was implemented by PaintOmics3 that allows integration of four types of omics data—gene-based, metabolite-based, region-based and regulatory omics to create pathway network where each node represents pathways and edges represent shared features or KEGG database connection [25]. The behavior of each pathway in a condition is summarized by pathway enrichment analysis on each omics data. Similar approach was also used in GraphOmics where transcriptomic, proteomic and metabolomic data are mapped to Reactome reactions and pathways based on their biochemical relationships [23]. Another multi-omics data integration is iOmicsPASS, which involves computations of interactions’ scores based on transcriptomic, proteomic and DNA copy number data for all possible interactions in a transcription factor regulatory network or PPI network [26]. The weighted network can then be used to identify sparse subnetworks predictive of specified phenotypes. Over the last decade, omics technologies have evolved from analysis of bulk tissue samples to single cell.

### Combining knowledge and data for network construction

Efforts have also been made to use the information in curated databases along with high-throughput experimental data for construction of networks. Pouryahya *et al.* [27] used known interactions from Human Protein Reference Database (HPRD) and gene expression data from GDSC to create weighted protein–protein interaction networks which they utilized for prediction of drug sensitivity in cancer cell lines. While this method focused on creating homogeneous networks, approaches have also been explored to create heterogeneous networks connecting different biological entities using both known interactions and interactions based on high-throughput data. For example, Scalable Precision Medicine Open Knowledge Engine (SPOKE) is a large heterogeneous network containing nodes of 11 types including drugs, diseases and genes, connected by 24 types of interactions sourced from existing knowledgebases and based on omics profiles like those of LINCS L1000 [28]. SPOKE was created with the aim to boost discovery of personalized therapeutics by linking electronic health records of individual patients to the different biological entities in the network. Lee *et al.* [29] created a multilayered heterogeneous network of SNP-gene disease to identify key genes in dementia. In their network, each layer consisted of a homogenous network of either SNP, gene or disease, and the three layers were connected based on interrelationships between them. The disease and gene network was based on existing data, while the SNP network was created by calculating interactions between SNPs from GWAS data.

Apart from creating heterogenous networks, knowledge and data can also be combined through similarity-based networks. For example, drug–drug networks can be created by calculating the similarity between their chemical structures [30] or their targets which in turn can be retrieved from databases like DrugBank. Similarly, disease–disease similarity networks can be constructed by measuring the degree of similarity between various diseases based on different criteria like matched MeSH terms in medical description of diseases [31], disease-gene associations [32, 33], comorbidity risk [33] and omics profiles. In a recent work, Jin *et al.* [34] utilized similarity network fusion to merge multiple drug–drug networks constructed based on known drug–drug interactions, similarities based on its association with proteins and side effects, omics profiles and structural properties which were then used to retrieve drug features to be used in a drug-disease heterogeneous network for predicting drug-disease associations.

Similarity networks offer a potential solution to combine information from different sources without much regard to the data structure. They have been particularly exploited to create the different layers in a multiplex network. Halu *et al.* [33] created a multiplex network of 779 human diseases by calculating the similarities between various diseases based on phenotypic and genotypic data with the aim of characterizing and subtyping diseases. More recently, Pio-Lopez [35] used network embedding on multiplex-heterogeneous networks for repurposing drugs for SARS-CoV-2. The network consisted of two separate multiplex networks of human molecular interactions and drugs connected by drug-target associations. Each multiplex layer in the network was created either by retrieving known interactions or based on similarity.

## NDM algorithms

**Figure 2:**
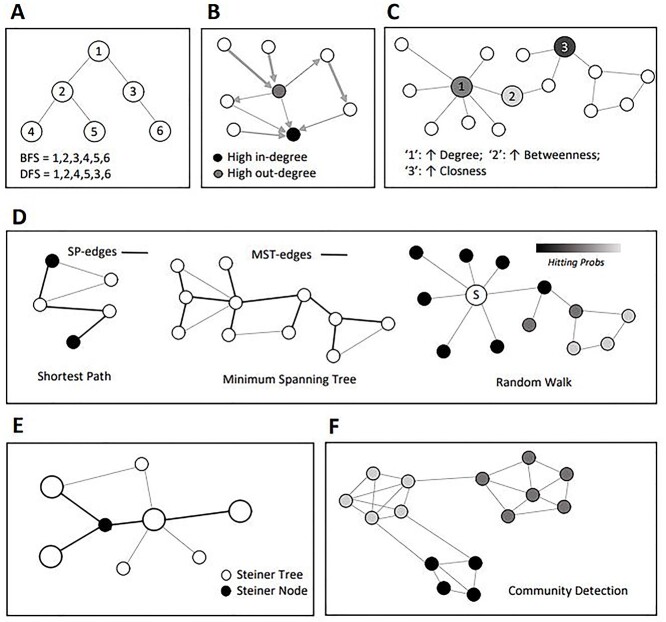
Basic algorithms in network data mining. (A) Examples of graph search algorithms. (B) Examples of in- and out-degree centrality. (C) Examples of nodes highlighting the main characteristic of different centrality scores. ‘1’ has a high degree; ‘2’ has a high betweenness score and ‘3’ has a high closeness score. (D) Network proximity with SP, MST and RW. (E) Example of a Steiner tree. (F) Example of CD in graph. The edges of the SP, MST or ST are presented in bold.

In this section, we briefly introduce basic NDM algorithms that are often applied to study the MoA of diseases and drugs (see Figure 2).

### Graph search algorithms

Exploring graphs means apply graph search algorithms, which can aim at general discoveries (e.g. to identify disease-deregulated genes that are close to a given drug target), or explicit search (e.g. to find the shortest path [SP] between a given disease-deregulated gene and a known drug target). Breadth First Search (BFS) [36] and Depth First Search (DFS) [37] are two NDM algorithms for traversing a graph, and they are often used as first step for many other NDM strategies. Figure 2A shows the order in which the nodes of a graph are visited when performing BFS and DFS.

### Centrality algorithms

Centrality algorithms help identify key players in biological networks. For example, it has been shown that highly connected vertices in protein interaction networks are often functionally important and the deletion of such vertices is related to lethality [38, 39]. There are three main centrality algorithms: Degree Centrality (DC), Closeness Centrality (CC) and Betweenness Centrality (BC). DC can be used to rank the nodes according to their degree, which corresponds to the number of edges linked to the node. For directed networks two-degree centralities exist: the in-degree centrality and the out-degree centrality (see Figure 2B). The CC instead uses information about the length of the SPs within a network; it compiles the sum of the minimal distances of a node to all other nodes. Then, BC measures the fraction of the SPs that pass through a node. It quantifies the ability of a node to be an important link between other nodes. BC could be used to improve drug targeting by finding the control genes for specific diseases. It has also been used to detect preferential targets of pathogen effectors [40]. Figure 2C shows examples of nodes with a high degree, betweenness or closeness centrality.

### Path finding algorithms

Path finding algorithms utilize graph traversing strategies to explore possible paths between nodes in a molecular network. There are four main path finding algorithms: *SP*, *minimum spanning tree (MST)*, *random walk (RW)* and *Steiner tree.*

The SP algorithms search for the cheapest path in terms of the number of hops or edge weights. Edge weights are often included in molecular networks to represent important information such as the co-expression level of two interconnected genes (a continuous weight). SPs can be found by using the Dijkstra’s Algorithm [41] which determines the path that minimizes the total distance (weight) between a given node (which is called the ‘source node’) and all other nodes in a graph. It should be noted that the weight, in this case, is assigned to the edge or connection between two nodes. Dijkstra’s Algorithm first identifies the lowest-weight relationship from the start node to directly connected nodes. It keeps track of those weights and moves to the ‘closest’ node. It then performs the same calculation, but now as a cumulative total from the start node. The algorithm continues in an iterative way by computing the same calculations and updating the cumulative weights. During the iterations, it will always select the lowest weighted cumulative path to advance along, until it reaches the destination node. SP algorithms have been extensively used to identify drug-target interactions [42–44].

Another interesting application of pathfinding algorithms is the one based on MST (Weight). A MST of a graph is a subset of edges that minimize the costs (or edge weights) needed to connect all the vertices together without using cycles. Greedy algorithms work well for computing the MST and there are two main implementations: the Kruskal’s [45] and Prim’s algorithms. MST can be used to identify key dysregulated genes in disease-specific gene and protein networks [46]. Moreover, MSTs are often applied to implement phylogenetic network analysis. For example, MST was recently used to derive a phylogenetic tree and measure differences in SARS-Cov-2 variants [47].

The RW algorithm [48] is used to verify which nodes are more frequently visited on a random path in a graph. It simulates a traversal of the graph in which the crossed graph edges are chosen at random. After repeating this process several times, it should then be possible to measure the node-to-node proximities. In a classic RW, each edge has the same, possibly weighted, probability of being selected, and this probability is not influenced by the previously visited nodes. The most used variant of RWs is called Random Walk with Restart. Figure 2D graphically illustrates examples of SP, RW and MST. The SP and MST edges are shown in bold.

Steiner tree algorithms solve a classical combinatorial optimization problem which focuses on identifying a subgraph of minimum cost connecting a given set of seed nodes. Figure 2E includes an example of ST. The input biological network must include edges with costs which correspond to confidence or frequency of that interaction, and prizes for the nodes, which could be linked to the measurements of differential expression. The set of nodes with assigned prizes are referred to as terminal nodes, while nonterminal nodes, interconnecting the nodes with a prize, are called Steiner nodes, which are represented as black nodes in Figure 2E. STs are a very powerful network-based tool. They have been used to study to understand the mechanism of complex diseases [49], to search for candidate drug targets in SARS-CoV-2 and to extract drug repurposing candidates [50].

### Community detection algorithms

These algorithms can find communities within a biological network (see Figure 2F). A community is a group of nodes that is tightly connected and, at the same time, the same nodes are loosely connected with every other node in the network [51]. There are many different techniques that can be implemented for detecting such communities. Two common techniques are label propagation (LP) [52] and the Louvain algorithm (LA) [53]. LP identifies communities by an iterative process where node labels are spread to the neighbors until convergence is reached, which means that nodes with the same label constitute a community. The basic idea of LP is that a single label can quickly become dominant in a densely connected group of nodes, but it will not be able to ‘dominate’ when traversing sparsely connected regions. On the other hand, LA applies a modularity-based strategy. It maximizes a modularity score for each community, where the modularity quantifies the quality of an assignment of nodes to communities. This means evaluating how much more densely connected the nodes within a community are, compared to how connected they would be in a random network. LA utilizes a hierarchical clustering algorithm that recursively merges communities into a single node and executes the modularity clustering on the condensed graphs. Table 3 includes a short description of research studies aiming at using NDMs for studying disease and drug effects upon different biological networks.

**Table 3:** Application of network data-mining algorithms for studying diseases, drugs and their associations

Algorithm	Target	Application	Description	Reference
*BFS + DFS*	Explore disease and drug effects	Network-based prioritization of gene-disease associations	BFS and DFS were used in combination to compute a low-dimensional vector representation for all nodes in a network. These vectors are then used for gene prioritization.	[130]
*BFS*	Explore disease and drug effects	Identify key and master regulatory genes in disease-specific gene network	BFS was applied to find a minimum set of connected genes such that every other gene in the network is one hop connected with a gene in this set (a minimum connected dominating set of genes).	[131]
*DFS*	Explore disease and drug effects	Identify connected subnetworks in disease-specific biological networks	DFS was extended to identify active gene (or protein)-based modules across multilayer networks (i.e. networks composed of different layers, where every layer is an independent network).	[132]
*Shortest Path*	Explore disease and drug effects	Mine associations between disease and drug targets	Shortest paths were used to compute network proximity of drug-disease pairs. These proximity scores are then evaluated for the prediction of effective drug combinations or adverse effects.	[73]
*Shortest Path*	Explore disease effects	Mine novel disease-associated genes	Shortest paths were utilized to detect genes that are closely related to known disease-associated genes.	[43]
*Minimum Spanning Trees*	Explore disease variants	Divergence analysis of disease variants	A phylogenetic network analysis of 160 complete human severe acute respiratory syndrome coronavirus 2 (SARS-Cov-2) genomes uses minimum spanning trees to build a phylogenetic tree and measure differences SARS-Cov-2 variants.	[47]
*Minimum Spanning Trees*	Explore disease variants	Identify patient-driven dysregulated networks	A modified Kruskal minimum spanning tree search strategy was implemented to determine the maximum dysregulated subnetwork for drug treatment in a cohort of cancer patients.	[46]
*Random Walk*	Explore drug effects	Predict drug-target interaction prediction by using multiple networks	The RWR algorithm was used to infer the cascading effect triggered by perturbed drug targets. The so-called ‘diffusion state’ is then used to compute prediction scores of drug target interactions.	[80]
*Random Walk*	Explore disease-drug relationships	Identify new indications for existing drugs	An integrated heterogeneous network was constructed by combining multiple sources including drugs, drug targets, diseases and disease genes data. Then, RWR was applied to rank diseases starting from known drug targets.	[30]
*Centrality*	Explore disease effects	Identifying gene-disease associations using centrality on gene networks	Text mining and network analysis were used in combination to build disease-specific gene-interaction networks and mine gene-disease associations on the basis of four node centrality scores: degree, eigenvector, betweenness and closeness centrality.	[133]
*Centrality*	Explore drug-disease relationships	Explore the SARS-CoV-2 virus-host-drug interactome for drug repurposing	A network was built to present viral-host protein interactions, host-protein interactions and drug-protein interactions. Then, betweenness and closeness centrality are used to rank and select known drugs targeting an optimized set including key viral and host proteins.	[102]
*Community Detection*	Explore drug-disease relationships	Identify disease-gene and drug-gene associations	A network including disease-drug interactions is built based on known disease-gene associations and drug targets. Then, modularity-based community detection algorithms were used to identify clusters of diseases and drugs that could suggest novel drug repositioning candidates	[134]
*Community Detection*	Explore disease effects	Identify phenotype-driven modules in gene networks	The Louvain algorithm is utilized to identify biologically relevant gene modules that change under different environmental conditions and biological states.	[135]

### Machine learning and graph embeddings

Biological networks can be transformed into vector space to allow machine learning algorithms to work with them and solve important supervised and unsupervised learning tasks for precision medicine approaches, such as biomarker discovery [54] and patient subtyping [55]. To this end, however, graph embedding strategies are often implemented. A graph embedding process aims at creating a lower dimensional representation of an entire graph, by preserving general properties of the graph structure. A more simplistic approach is to use connected feature extraction strategies, where the objective is to extract specific features from the analyzed biological networks. For instance, we could define features representing connection-related metrics, such as the number of relationships going into or out of nodes, a count of potential triangles, and neighbors in common. Moreover, NDM algorithms, such as community detection (CD), and network diffusion, can also be used to extract graph-based features [56]. RW and, more in general, network propagation methods are commonly used for graph embedding [57–59]. Graph embedding is often applied to implement link prediction strategies in drug discovery, such as identify drug-target interactions [60], drug–drug interactions [61] and drug-related side effects [62].

## NDM in drug discovery

Here we describe computational methods that utilize NDM for addressing drug discovery–related problems such as the identification of drug-targets, or drug-disease associations, drug combination and drug sensitivity prediction. Table 4 reports key information for each tool, including the availability of the software implementing the proposed methods, and a summary of their key properties and limitations, which covers the aspects of scalability and computational complexity.

**Table 4:** Comparison of NDM-based approaches used in drug discovery. The methods are grouped based on four categories: basic NDM algorithms network propagation and random-walk based methods (which do not rely on graph embeddings), matrix factorization and graph neural network-based methods

Software	Algorithms	Objectives	Input data	Key properties	Limitations
*Pure network-based methods*					
Guney’s toolbox	Network proximity between drug targets and disease modules	*In silico* screening, drug repurposing, drug combinations	PPIs, DTIs, disease gene sets	Estimates drug efficacy and safety in gene interaction network context. Uses network-based proximity measures for the discovery of drug combinations. Low computational complexity after computing the shortest paths between all proteins. Estimation for each drug or drug combination is independent of each other and hence it should be highly scalable. Time complexity is not specified, but it should be bounded by *O(N + E log N)*, since it is essentially based on the calculation of shortest paths.	To yield accurate results the set of interactions and disease associated genes should be accurate and complete. Currently focused on only PPIs.
GPSnet	Network proximity, gene module detection based on greedy algorithm with multiple random initializations	*In silico* screening, drug repurposing	Patient mutation and gene-expression data for disease modules, DTIs (combined from 6 sources), PPIs (combined from 15 sources), drug-induced transcriptome data for GSEA	Uses cancer-type-specific omics data for disease module detection and cancer specific efficacy estimation based on network proximity. Uses GSEA and cell line expression data to confirm whether disease genes are up-/downregulated by drugs Identifies omics-driven disease gene modules (or pathways). Has low computational complexity and high scalability.	To yield accurate results the set of interactions and disease associated genes should be accurate and complete. Currently focused on only PPIs. Benchmarking analysis based on identified disease modules, but there is no accuracy estimated from known drug-disease associations.
SAveRUNNER	Network proximity, drug-disease module detection	*In silico* screening, drug repurposing	PPIs, DTIs, disease gene sets	Estimates drug efficacy using gene interaction networks and network proximity. Prioritizes associations between drugs and diseases located in the same network neighborhoods. Identifies off-label of drugs to be repositioned. Time complexity is not specified, but the module detection algorithms runs in near linear time, *O(n log^2^ n*) and network proximity should be bounded by shortest paths computation, i.e. *O(N + E log N)*. High scalability.	To yield accurate results the set of interactions and disease associated genes should be accurate and complete. Low benchmark accuracy.
ThETA	Dijkstra’s algorithm, node centrality based on degree, clustering coefficients and betweenness	Target prioritization	PPIs, tissue-specific gene-expression, disease gene sets	Drug target efficacy and safety estimation with network topology measures. Computes tissue-specific efficacy estimates. Time complexity is bounded by *O(n^3^)* where *n* is the number of proteins*.*	It depends on the accuracy of known disease-gene associations. The computation of betweenness centrality is expensive for large networks.
MeTeOR community detection	Recursive Louvain method	Find disease- and drug-specific pathways	MeSH term co-occurrence in literature	Identifies novel disease and drug specific biological pathways from genes contained in the same communities. Efficient module detection via Recursive Louvain (RL) method. Requires computationally expensive text mining to produce the network in the first place. Might scale poorly with larger networks due to recursiveness. Time complexity is not specified.	The power of the method for drug discovery depends on the amount of literature that is already published on the topic of interest.
*Network propagation and random-walk-based methods*					
MBiRW	Bi-random walk with restart	Drug repurposing	Drug structure for molecular fingerprints, MeSH terms of diseases, known drug-disease indications	Simultaneous random walks on drug similarity network and disease similarity network. Known associations are systematically used to adjust similarities and as seed node sets for random walk with restart. Applies clustering analysis to further increase similarity of drugs and diseases belonging to the same cluster. Time complexity is not specified, but it should approximately be cubic in the number of nodes *O(n^3^)*.	Repeated adjacency matrix multiplication scales poorly when the number of drugs and diseases becomes very large. Additional biological information could be used to further improve similarities.
DrugNet	Network propagation	Drug repurposing	Drug annotations, disease ontology, known drug-disease indications, DTIs, PPIs, disease gene sets	Able to integrate data from complex networks involving a wide range of types of elements and interactions The algorithm can work with an arbitrary number of heterogeneous data networks. Computational time relying on network propagation within a network—*O(n^3^)*; and across the network—*O(n^2^)*. Time complexity should approximately be bounded by *O(m × n^3^)*, where *m* is the number of networks and *n* is the number of nodes.	Network completeness significantly affects the quality predictions. The authors identified issues stemming from network topology with certain categories of drugs. Low scalability.
*Matrix factorization-based methods*					
SCMFDD	Similarity constrained matrix factorization	Drug repurposing	Drug-disease associations, drug molecular structure, drug target proteins and enzymes, drug pathways, drug–drug interactions, disease MeSH DAGs (used for semantic similarity)	Predicts drug-disease associations by using matrix factorization (MF). Improves MF by using similarity constraints to account for the biological context. Simple and efficient algorithm (should scale better for larger networks than MBiRW) Time complexity is not specified, but it should be approximately bounded by *O(n^2^ + m^2^ + nmk^2^)*, where n * m is the dimension of the matrix of observed drug-disease associations and *k* is the number of hidden dimensions.	Three hyper-parameters need to be tuned. Drug similarities were not integrated, but rather tested separately. Prediction performance was not much higher than those of previously published methods.
NMF-DR	Non-negative matrix factorization, similarity network normalization and fusion	Drug repurposing	Known drug-disease indications, drug similarities and disease similarities collected from four previous studies based on: drug clinical annotations, drug structure, drug targets, drug pathways, drug–drug interactions, disease MeSH DAGs, disease ontology, disease gene sets	Novel approach for constructing a heterogeneous network of drugs and diseases. Aggregates and normalizes similarities between drugs and diseases from different data sources by using similarity network normalization and fusion (SNNF). Provides an improved non-negative matrix factorization (NMF) method which is based on a rank selection method and singular value decomposition (SVD) methods. Could be applied to other bi-partite networks with any number of similarities. Time complexity is not specified.	The matrix initialization cost (using SVD) was identified as a bottleneck by the authors. The similarity fusion step can lose some complementary information from different similarities even though it showed an improvement over the individual similarities that were tested separately.
MSBMF	Matrix factorization	Drug repurposing	Drug-disease associations, drug clinical annotations, drug structure, drug side effects, drug–drug interactions, drug targets, disease MeSH terms, disease ontology	Uses matrix factorization to decompose the drug-disease association matrix into a drug-feature matrix and a disease-feature matrix. Multiple drug- and disease-based similarity constraints are used without applying a fusion step. Latent features are then extracted to infer missing drug-disease associations. Could be applied to other bi-partite networks with any number of similarities. Time complexity is not specified.	It utilizes non-convex optimization which can get stuck in local optima.
DTINet	RWR, Diffusion Component Analysis (DCA), inductive matrix completion	Drug-target prediction, drug repurposing	DTIs, drug–drug interactions, drug-disease associations, drug-side effect associations, disease gene sets, PPIs, drug molecular structures, protein sequences	Integrates diverse information from heterogeneous networks. Uses diffusion component analysis (DCA) to embed nodes of several networks between drugs, targets, side-effects and diseases to extract topological drug and target features. Uses inductive matrix completion to find projection of low-dimensional drug and disease representation such that known drug-disease pairs are geometrically closer in the mapped space. Can be applied to other bi-partite networks with any similarity or association related to drugs and proteins Time complexity is approximately bounded by O(k_1_ * n^3^ + k_2_ * n^3^ + n), where *k_1_* and *k_2_* are, respectively, the number of drugs and target-based similarity networks.	Using network propagation for graph embedding is no longer state-of-the-art. A set of negative training examples is required for the matrix completion method used and in practice they are sampled from the set of unknown predictions, which can cause bias in the predictions.
*Graph neural network-based methods*					
NeoDTI	Deep learning, graph convolutional networks	Drug-target network completion, drug repurposing	DTIs, drug–drug interactions, drug-disease associations, drug-side-effect associations, disease gene sets, PPIs, drug molecular structures, protein sequences	Predicts drug-target interactions by using several heterogeneous networks between drugs, side effects, proteins and diseases. Embeds nodes in heterogeneous networks by using neighborhood aggregation. Implements end-to-end DTI prediction based on deep learning. Uses multi-objective optimization aiming to preserve topology of input data via reconstruction. Comparatively high predictive performance (improves DTINet). Could be applied to other bi-partite networks with any similarity or association related to drugs and proteins. It can handle large-scale data. Time complexity is not specified.	Computational cost of deep learning can be high, although generally it scales well with more data. Needs to sample negative examples randomly from unknown associations.
DeepDTnet	Deep learning, stacked denoising autoencoder, positive-unlabeled matrix completion	Drug-target network completion, drug repurposing	DTIs (assembled from 6 sources), drug molecular structure, PPIs (15 sources), drug–drug interactions (1 source), known drug-disease indications (3 sources), drug side effects (4 sources), protein sequences, tissue gene-expression, gene ontology, drug annotations, disease gene sets (3 sources)	Predicts drug-target interactions by using multiple heterogeneous networks between drugs, side effects, proteins and diseases. Node embeddings used as drug and target features are extracted by DNGR which uses random surfing, positive pointwise mutual information and stacked denoising autoencoders. Positive unlabeled matrix completion addresses sparsity of associations and lack of negative examples in drug-target graph. Could be used to integrate any weighted networks related to DTI or other bi-partite link prediction. Higher predictive performance compared to DTINet. Time complexity is not specified.	Computational cost can be high for learning multiple deep autoencoders. The authors indicate that their classification of DTI based on a weak binding affinity cut-off might risk false positives. Predictions were slightly biased against drugs with lower average similarity to other drugs, but still very good.
HeTDR	Deep learning, similarity network fusion (SNF), sparse autoencoders, text mining, representation learning for attributed multiplex heterogeneous network	Drug repurposing	Drug-disease associations, drug–drug interactions, DTIs (3 sources), drug-side effect association (4 sources), drug molecular structure, drug clinical annotations, protein sequence similarity, gene ontology (GO), drug similarities (structure, clinical annotation semantics, target similarity, 3 GO-based semantics), text-mining-based disease features	Utilizes a combination of autoencoder, text-mining and deep learning-based link prediction. Sparse autoencoder extracts drug attributes from multiple similarity measures fused together with SNF. Text mining is used to extract rich disease attributes. Link prediction method (GATNE-I) utilizes attributed heterogeneous network graph embedding. GATNE-I could perhaps be used to integrate multiple relations between drugs, diseases and other entities. Text mining could be applied to other corpora. Significantly outperformed other methods in comparison. Time complexity is not specified.	Uses three separately trained deep learning methods with significant computational cost. The final network features only drugs and proteins and could perhaps be improved by adding relevant biomolecules such as proteins.
MGRL	Deep learning, graph convolutional networks, node2vec, random forest classifier	Drug repurposing	Drug-disease associations, drug molecular structure, DTIs, disease MeSH DAGs (used for semantic similarity)	Combines neighborhood attribute aggregation and non-attributed graph node embedding. Uses graph convolutional networks for feature extraction by embedding drugs and diseases based on their immediate neighborhood and attributes. Uses node2vec to embed drugs and diseases in the global network context. Uses random forest for predictions. Time complexity is not specified.	It uses a simplified version of graph convolution that may not learn rich representations of the nodes. Nevertheless, the computational costs of deep learning are typically higher than other methods. The prediction performance was only slightly higher than previous deep learning methods.
GRLMN	Deep learning, stacked autoencoders, Large-scale Information Network Embedding (LINE), random forest classifier	Drug repurposing	Drug-disease associations, drug molecular structure, drug target proteins and enzymes, drug pathways, drug–drug interactions, disease MeSH DAGs (used for semantic similarity), disease and drug associations for miRNA, lncRNA and proteins, DTIs, disease gene sets, PPIs	Integrates association networks consisting of multiple biomolecules. Uses heterogeneous network node embedding for drugs and diseases as well as a drug fingerprint autoencoder for feature extraction. Uses random forest for predictions based on node embeddings and attributes. Could be used to incorporate relations among any number of different entities. When testing different embedding algorithms, LINE outperformed node2vec slightly. Time complexity not specified, but LINE is approximately bounded by *O(dKE)* where *d* is average degree, *K* is number of negative examples and *E* is number of edges.	When updating the input network, the whole model needs to be retrained, although the computational cost is not very high according to the authors. Low improvement over SCMFDD prediction performance.
Decagon	Deep learning, graph convolutional networks, tensor factorization	Prediction of side effects for drug combinations	PPIs (assembled from 4 sources), DTIs, drug side effects (2 sources), drug–drug combination side-effects	Predicts polypharmacy side effects. Represents side effects for drug combinations as different drug–drug relations which represent different side effects. Uses end-to-end deep learning combining a graph convolutional network as an encoder of drug–drug and drug-protein relations as well as tensor factorization as a decoder to predict drug combination side effects. Significantly outperformed other multi-relational link prediction methods in comparison. Time complexity is not specified.	It samples random missing links for negative examples in training. Side effects can depend on the dose and patient, which cannot be accounted for by using this method.
*Methods for drug sensitivity and synergy prediction*					
CDCN	Network model	Drug sensitivity prediction	Drug response data (IC50) in different cancer cell lines, cell line gene expression at steady state, drug molecular structures	Constructs a heterogenous network of drugs and cell lines, where drug-cell line edges are weighted by drug response, and drug–drug and cell line-cell line edges are weighted by their similarity. Predicts drug response for new drug on new cell line by using a network model. The predictions are optimized by evaluating a network model over a range of decay parameter values. The optimization algorithm’s time complexity is bounded by the square of the number of drug-cell line pairs which could become an issue with larger datasets.	The suggested strategy for predicting responses in patients requires that there exists a correlated gene-expression profile for which drug response data is known at least for some drugs; however, in practice drug response is typically analyzed in cell lines which often do not exhibit strong correlation with patient profiles.
Pouryahya’s method	Network model, clustering, random forest regressor	Drug sensitivity prediction	Drug response data (IC50) in different cancer cell lines, cell line gene expression at steady state, PPIs, cancer associated gene set, drug molecular structures	Uses a stochastic message-passing process to define an invariant measure of gene expression in a PPI network. Uses Wasserstein distance and optimal mass transport to calculate distance between cell line gene expression profiles. Predictions were made with random forests based on gene expression and drug molecular features. Cell lines and drugs were clustered and separate regression models were trained on different cluster pairs. Time complexity is not specified.	Predictions depend on clustering information, assigning drugs and cell lines to their nearest cluster works as long as they are close to existing clusters, but might cause issues otherwise. Limiting analysis known cancer genes may discard useful information. Need to learn one model per drug-cell line cluster pair.
PaccMann	Network propagation, deep learning, multimodal attention-based neural networks, recurrent neural networks, convolutional neural networks	Drug sensitivity prediction	Drug response data (IC50) in different cancer cell lines, cell line gene expression at steady state, drug molecular structures, PPIs	Uses network propagation to extract subset of informative genes that is used as input in an end-to-end neural network to predict drug sensitivity. Attention-based neural networks offer better interpretability compared to other deep learning approaches. SMILES-based feature extraction outperformed molecular fingerprints. Time complexity is not specified.	The authors mention that adding gene expression profiles from healthy cell lines could improve the extraction of cancer specific features. The method uses PPI for selecting genes, but otherwise does not integrate biological networks.
DeepCDR	Deep learning, graph convolutional networks, convolutional neural networks, feed-forward neural networks	Drug sensitivity prediction	Drug response data (IC50) in different cancer cell lines, drug molecular structure, cell line multi-omic profiles (mutation, transcriptomic, epigenomic), patient multi-omic profiles and clinical annotations (used for validation only), known cancer associated gene set	Uses a uniform graph convolution network (UGCN) to extract rich drug representations from 2D drug graph structure. Integrates multi-omic data to characterize cell lines. Based on ablation analysis epigenomic profiles were particularly useful. Significantly outperformed other baseline machine learning models. Time complexity is not specified.	Does not integrate biological networks. UGCN uses fixed size inputs by utilizing a complementary drug structure graph that is used to complete the adjacency matrix.
Y Guan’s method	Network propagation, random forest regressor	Drug synergy prediction	Monotherapy and drug combination response data (IC50) and synergy scores in different cancer cell lines, DTIs, multi-omic cell line profiles (mutations, transcriptome, epigenome), gene network	Uses network propagation to simulate post treatment molecular profiles for drug combinations in each cell line. The simulated profiles were used as features for random forest-based regression. Trains ensemble of models restricted to subsets of all, one or two drugs for training. The monotherapy data was found more informative than simulated post combination treatment features, but still provided additional value for predictions. Time complexity is not specified.	The local synergy model trains one classifier for each drug combination, which does not scale well for a large number of drugs, although could still be reasonable by choosing an efficient method or appropriate hyper-parameters.
PRODeep Syn	Deep learning, graph convolutional network, matrix completion	Synergistic drug combination prediction	Drug combination response data (IC50) and synergy scores in different cancer cell lines, drug molecular structures, cell line gene expression and mutation profiles, PPIs, gene sets used to define node attributes (position, motif, immunological signature)	Merges PPIs, gene annotations and cell line gene expression to represent cell lines. Performs attributed graph node embedding with GCN to extract features for predicting cell line-specific gene expression profiles from PPI and gene annotations. The learned cell line hidden state and drug features are used in a fully connected deep neural network to predict drug synergy. Time complexity is not specified.	The authors noted that the predicted synergies tended to be lower than observed for drug combinations with high synergy scores due to potential issues with low synergy scores being very common in the training data. The performance improvement over other deep learning methods was quite low.
Dcombo Net	Heterogenous network RWR	Synergistic drug combination prediction	Known synergistic drug combinations, drug annotations, drug molecular structure, drug side effects, DTIs, drug-related pathways, PPIs, cancer pathways, baseline and drug exposed cell line gene expression profiles	Predicts anticancer drug combinations. Uses random walk in a heterogeneous network of drugs, genes and pathways. The walker jumps between subnetworks depending on jumping probability and the subnetwork. Can predict sample-specific drug combinations by modifying the network based on gene expression. Time complexity is not specified, but the main random walk algorithm is approximately bounded by O(n^3^) where n is the number of nodes in the heterogeneous network.	Three different jumping probabilities need to be optimized. Performance was evaluated by classifying drugs into two or three groups (combinable, uncombinable, intermediate). Drugs with unique mechanisms that are relatively disconnected from the rest of the network are less likely to be predicted well.

Computational methods can support fundamental stages of the drug development process, including the identification of putative drug targets, the *in silico* screening of drug compounds and drug combinations for the treatment of diseases. *In silico* screening can not only prioritize drugs for in vitro testing but also aid in drug repurposing [63]. These steps can lower the cost of drug discovery significantly, by evaluating efficacy and safety aspects of drugs or drug targets. A basic principle in computational drug discovery is that the best drugs should target the disease associated genes (or proteins) as directly as possible. However, not all genes/proteins are targetable and often we are working with a limited set of known drugs so the distance between drug targets and disease genes can be used as a rough estimate of the potential efficacy of drugs while considering the network context. These networks can be used to identify novel drug targets and new use for existing drugs by using proximity-based efficacy evaluations [64]. Pre-efficacy evaluations can also be made by considering how well drugs are able to reverse or target these disease perturbations. Safety is also of high concern, and it can be assessed for both putative drug targets and known drugs, especially when considering drug combinations which can induce large perturbations in biological systems. Network approaches allow the examination of the local properties and topology of drug targets, such as node centrality, which are key factors contributing to side effects and should be taken into consideration in rational drug design [65]. For heterogeneous networks, such as those linking disease-perturbed genes to other biological entities (e.g. pathways, known drug adverse reactions), safety can be more carefully addressed by assessing whether drug targets are linked, for instance, to cancer hallmark pathways or known drug-ADR properties.

Due to the biological relevance of the properties of gene networks, topological measures have been widely adapted for drug-repositioning to uncover functional relationships between drugs and diseases [66]. Particularly popular are the network-based proximity approaches [64, 67–71], which use shortest distances in biological networks to estimate efficacy and safety of drugs given a gene set corresponding to their targets and disease gene modules in a PPI network. Typically, these methods define network proximity as the average distance of each drug target to their closest disease gene and normalize them based on the network proximity of random gene sets. They have been applied broadly to many diseases such as cancer, cardiovascular disease and COVID-19. SAveRUNNER [72] is a novel network proximity-based tool for drug repurposing. It enhances the typical average-closest-distance-based z-score with a weight derived from clusters computed from network-proximity-based similarity. Drug and disease clusters are computed by using a greedy optimization of network modularity. A quality metric is computed for each cluster and then used to adjust the drug-disease similarities which are finally normalized with a sigmoid function. The final similarities were then exploited to prioritize drugs for repurposing. Another recently published network proximity-based method is GPSnet [73]. It targets specific disease gene modules derived from integrating patient DNA and RNA profiles with a PPI network. The modules are initialized randomly and extended based on several criteria including PPI connectivity significance, gene co-expression significance and mutation frequency. The identified disease modules are then used in the GPSnet targeting algorithm which combines network proximity of known drug-target genes to module genes and gene-set enrichment analysis (GSEA) based on drug-induced transcriptome data from ConnectivityMap [16]. This method could be applied to subtype-specific modules within a given cancer disease to identify repurposable drugs for precision medicine. However, the results are highly dependent on the data used to infer disease modules and consequently the approach requires a large enough dataset to infer sufficiently relevant gene co-expression networks and mutation profiles. On the other hand, these datasets do not need to consist of the same set of patients, i.e. if the DNA and RNA profiles represent the same disease (sub)type, the disease module inference can utilize different datasets. An important advantage of proximity-based methods is that the efficacy estimation does not depend on other drugs or diseases and similarities between them. However, the quality of estimates greatly depends on the accuracy of drug and disease associated gene sets. Furthermore, predictions can be sensitive to missing links in the network.

Network topology measures can also be used to rank drug targets. ThETA [44] estimates the efficacy of target-genes in treating diseases by utilizing tissue-specific gene co-expression networks and gene-expression data. It also estimates safety based on network centrality measures as well as ADR and onco-driven scores [43]. Efficacy and safety estimates of target-genes can be used to prioritize the development of new drugs targeting the most efficacious and safe genes and are also applicable to drug repurposing. In addition to topology measures, basic algorithms such as module detection can be applied to discover relations in heterogeneous networks. MeTeOR-RL [74] is a literature-driven NDM approach in which the authors used CD algorithms to extract cluster of heterogenous biological entities from a heterogeneous network, namely MeTeOR. The network links Medical Subject Headings (MeSH) terms of genes, diseases and drugs that co-occurred in publications, and the communities can include both drugs and diseases as well as sets of shared genes. The authors aimed to efficiently synthesize pathway information from literature-mined associations between gene terms, and to verify whether these de novo pathways could link diseases with known drugs. Such a network can also support disease gene discovery and drug repurposing through novel predictions of disease- and drug-specific mechanisms. Interestingly, the authors observed that genes in drug-specific communities were enriched for genes up-/down-regulated by the corresponding drugs, by using the gene expression profiles from the LINCS database. Furthermore, successful cases of repurposed drugs were confirmed. More interestingly, diseases sharing communities had high comorbidity with each other and drugs sharing communities had many common side effects, consistent with related mechanisms. This confirms that when searching for drug combinations, selecting drugs from different communities can decrease the risk of adverse drug reactions [42].

RW is another powerful algorithm often used in network-based drug-discovery. For example, MBiRW [75] uses an alternating bi-RW algorithm with restart between a bipartite drug-disease network as well as drug–drug and disease–disease similarity networks to predict new drug-disease associations for drug repositioning. The authors defined drug similarities based on molecule fingerprints and normalized Tanimoto similarity based on randomly shuffled molecules. They then applied CD on the drug similarity network and use prior interaction information to adjust the network such that drugs belonging to the same cluster were more similar. The bi-RW algorithm was used to run two RWs with restart in parallel, one on the drug–drug network and one on the disease–disease network where the walks are initialized by the known drug-disease indications and the steps are averaged between the walks. Higher affinities were finally used to infer probable drug-disease indications. DrugNet [76] is another example of network diffusion algorithm for mining drug-disease and disease-drug prioritization through heterogeneous networks. DrugNet utilizes a NDM algorithm that exploits paths across different network domains (e.g. genes, disease, proteins) to define distances between queried sets of nodes (e.g. diseases) and nodes available in different network domains (e.g. drugs). These paths can then be used to perform drug-disease and disease-drug prioritization. RW methods are in principle highly scalable, although the scalability also depends on the implementation of the RWR which could determine different accuracy and scalability results. An alternative approach to RWR is that based on matrix factorization (MF).

Matrix factorization methods can be applied to drug-disease network completion where the known associations are approximated with a product of two low-rank matrices sometimes called feature matrices. The approximation can be used to predict new associations, e.g. a completed bi-partite drug-disease graph adjacency matrix can be used for drug-repurposing. SCMFDD [77] uses matrix factorization on drug-disease associations with drug and disease similarity-based regularization to bring similar drugs and diseases closer in the low rank spaces. Recently developed methods such as NMF-DR [78] and MSBMF [79] focus on integrating drug–drug and disease–disease similarities from multiple sources. NMF-DR first integrates disease and drug similarities individually, and then applies an improved nonnegative matrix factorization-based method to score unknown drug-disease pairs. On the other hand, MSBMF includes separate similarity constraint terms for each similarity type in the objective function for matrix completion instead of fusing multiple similarities into a single similarity matrix. Then, there are methods that focus on predicting drug targets for known drugs. For example, DTINet [80] integrates various networks, such as drug–drug or protein–protein interactions and similarities as well as drug-disease and protein-disease associations by using RWR and diffusion component analysis to obtain low-dimensional matrix representations (graph embeddings) of drugs and proteins. It then uses inductive matrix completion to find the optimal projection from drug representations to protein representation space such that the mapped features of drugs are close to proteins that they are known to interact with. The matrix completion step is very similar to matrix factorization approaches, although predictions for novel DTIs are based on distances between drugs and proteins in the mapped space rather than the completed matrix. The graph embeddings in DTINet are learned in an unsupervised manner separate from the prediction. A disadvantage of this approach is that the unknown drug-target interaction is treated as the negative training set. However, this set is noisy, as it may include novel, unknown drug-target interactions in itself.

New trends in network-driven drug discoveries also include graph neural network-based approaches. For example, NeoDTI [81] implements an end-to-end neural network to learn the embeddings via graph convolutional networks and uses a topology a preserving objective to learn to reconstruct the weights of the input graph. The network can then be queried for drug-protein pairs to predict their interaction. DeepDTnet [82] is very similar to DTINet except that it uses deep neural network architecture for learning graph embeddings and positive-unlabeled matrix completion to find the best projection from the drug space onto target (protein) space, such that the projected feature vectors of drugs are geometrically close to the feature vectors of their known interacting targets. New targets are the inferred by geometric proximity to the projected feature vector of the drug in the projected space. An interesting advantage provided by the DeepDTnet method, with respect to previously developed methods, is that unobserved drug-target associations are not treated as negative examples.

Most existing methods utilize a few data sources to represent disease similarities. To include further disease-related features, HeTDR [34], a newly developed method, utilizes biomedical text mining [83] to elaborate an extensive set of disease features which improves the accuracy of drug-disease associations’ prediction. HeTDR also uses network affinity propagation in combination with spare autoencoders [84] to derive drug-related features. Then, drug- and disease-related features are used as node attributes in a drug-disease network that is then embedded using GATNE-I [85], a deep learning-based approach for multiplex heterogeneous network embedding and link prediction by using edge and node embeddings with node attributes. These three elements are trained separately such that the drug and disease features are extracted without using drug-disease association data (other than text in disease-related literature). HeTDR combines many state-of-the-art methods to improve heterogeneous-graph-embedding-based drug repurposing. A major advantage of these methods is the possibility to include any number of node and edge types and to predict any type of interaction. A disadvantage that is shared with all link prediction methods is that predictions depend on the completeness of the interaction graph and similarity information used for learning which can significantly impact predictions for novel drugs or targets [81].

Another interesting niche of network-driven methods is that based on graph convolutional networks (GCNs), which are quickly becoming one of the most widely adopted deep learning architectures in drug discovery [86]. GCN architectures can support various types of networks from simple networks to attributed multiplex heterogeneous networks. These methods can be applied to link prediction as well as node and graph classification or to learn representations for small molecules. One of the advantages of GCNs is that they can learn directly from the network, and therefore there is no need to extract relevant features separately. However, GCNs often include a high number of parameters that need to be trained, i.e. overparameterization can be a major issue especially with heterogeneous and multiplex/multi-relational networks that must handle different types of nodes and edges. COMPGCN [87] aims to solve this issue for multi-relational networks by using linear combinations of basis embedding vectors.

Many methods use graph embeddings from GCN, RW or MF to extract features for a standard supervised machine learning classifier. For example, MGRL [88] uses GCNs to extract features for drugs and diseases from attributed bi-partite networks containing drugs and diseases from CTD [89]. Disease attributes were based on MeSH terms and drug attributes on molecular fingerprints. They also apply RW to separately embed nodes in the drug-disease graph. Lastly, they apply a random forest classifier to predict drug-disease associations based on the extracted features. GRLMN [90] integrates associations between drugs, diseases, proteins, lncRNA and miRNA and PPI. The nodes were attributed with heterogeneous features such as fingerprints, MeSH terms and nucleic acid sequences. Then, a graph embedding algorithm is used to extract node features that are subsequently used to train random forest-based classifiers to predict drug-disease associations.

Many existing network-driven methods for identifying drug-target or disease-drug associations do not adequately address safety aspects. Indeed, there are a few methods that try to identify side effects that may be liked to novel drug targets or drug combinations. A very interesting method that has recently been developed is Decagon [62], which utilizes graph convolutional neural networks and heterogeneous networks to predict adverse drug reactions (ADRs) for drug combinations. Decagon integrates PPI, DTI and DDI networks where different link types are used to represent different side effects of drug pairs. It uses graph convolution to represent nodes in a low-dimensional hidden layer which is used as input to a feed forward neural network that predicts ADRs. End-to-end neural networks can also achieve higher accuracy than two-stage models since the learned intermediate representation should be optimized for the task. The largest downside is perhaps that deep learning requires a large data set to learn model parameters that yield relevant latent representations and generalizable predictions. The method also cannot predict adverse reactions for combinations of novel drugs that do not have known adverse effect profiles and might perform poorly for newer drugs with fewer known side effects.

Network-based approaches have also been used to predict drug sensitivity and synergy. Synergistic drugs have potential to kill tumor cells more effectively while requiring lower doses; however, sensitivity and synergy are cancer specific, which makes drug combination particularly challenging. CDCN [91] builds a cell line-drug complex network in order to model drug sensitivity based on a weighted average of all other known sensitivities with weights that are based on drug and cell line similarities. The similarities were based on Tanimoto coefficient on drug features and correlation of cell line gene-expression profiles. Another novel method, here indicated as Pouryahya’s method, utilized stochastic processes and optimal mass transport (OMT) to compute distances between cell line gene-expression profiles in a PPI network and between drugs in a similarity network based on drug features. They clustered cell lines and drugs and then used random forest (RF) to predict drug sensitivity from the original gene-expression profiles and drug features, while training different RF for different cell line and drug cluster pairs. They showed that their distance measure could be used in CDCN to improve predictions and that the clustered RF model performed even better [27]. PaccMann [92] is another method for drug sensitivity prediction based on the use in combination of a PPI network, cell line gene expression profiles and deep learning. The PPI was used to reduce the set of genes by using RWR from known drug targets and selecting top 20 genes for each drug. They tested various deep learning architectures for encoding strings and compared to engineered fingerprints, showing that their model could improve upon them. Due to their availability, SMILES can be more convenient to use in encoders, but 2D graph representations of drugs are much richer and can be extracted with GCNs as demonstrated by DeepCDR [93].

The AstraZeneca-Sanger DREAM challenge also demonstrated the importance of using NDM algorithms for computational prediction of cancer drug combinations in a pharmacogenomic screen [94]. Indeed, the winning method, here indicated as Y Guan’s method, predicted drug response synergy in cancer cell lines by using network propagation in heterogeneous networks to extract informative features for machine learning–based prediction [95]. They used monotherapy data and approximated effects based on baseline molecular data, a gene interaction network, drug target information and network propagation-based features to train an ensemble of random forest classifiers focusing on different subsets of data.

PRODeepSyn [96] is a recently published method that applies GCN to embed cell lines based on omics data and a PPI network and then predicts drug-combination synergy based on the embedding and drug features. A novel idea proposed by this method is to learn a low-dimensional latent representation of cell lines which is based on optimizing a projection to the omics profiles associated with cell lines. The GCN learns to produce a projection matrix from PPI and node attributes from various gene sets thereby integrating prior biological knowledge to the learning of the latent features. Drug features and cell line latent representations were then exploited in a fully connected (FC) feed forward neural network (FFNN) to predict synergies. Their ablation study showed a minor improvement associated with the knowledge integration when compared to learning the projection directly, analogous to matrix factorization methods, or by using autoencoders. This method, along with other deep learning approaches for synergy prediction, outperformed common supervised machine learning methods. However, it should be considered that often training data sets for drug-combination prediction are very sparse. RW has also been applied to predict synergistic drug combinations, e.g. DComboNet [97], which integrates five generic networks, including known drug–drug, drug-gene, gene–gene, drug-pathway and pathway-pathway association networks. DComboNet can also address the construction of sample specific networks from gene expression profiles in drug-exposed and baseline cell-lines, which can be used to implement personalize medicine-based strategies for drug combination prediction. A major flaw in these drug sensitivity studies is the use of summary metrics, such as IC50, as the target instead of the whole response curve over a range of doses. It has been shown that predicting the whole response curve improves prediction quality also in terms of summary metrics [98]. Predicting responses for different doses also provides better translational utility since it can be used to identify the ideal dose that is maximally effective while minimally toxic. In addition, tensor factorization and random forests have also been applied to predict dose response matrices for drug combinations [99]. Integration of biological knowledge in network-based methods has been shown to improve over pure machine learning methods, so perhaps they can also be used to improve whole dose-response prediction as well.

## Drug repurposing for COVID-19 with NDM strategies

The emergence of SARS-CoV-2 caused a surge of interest in drug repositioning to treat COVID-19 [100]. Network-based strategies such as network proximity have been applied by various researchers [68–71]. For example, a novel method called SAveRUNNER [69] was applied to COVID-19 drug prioritization. It uses proximity for drug-disease prioritization while also weighing drug-disease pairs by using clustering such that pairs with target sets and disease gene sets in the same cluster are preferred. In the absence of *in vitro* validation, other computational estimators such as gene set enrichment analysis (GSEA) based on drug gene-expression signatures in human cell lines acquired from the Connectivity Map database and COVID-19-related transcriptomic datasets were typically used to see if the drugs could reverse the disease expression pattern [69–71] similarly to the method described by Sirota *et al.* [101].

In addition, large population data from COVID-19 registries have been applied to validate predictions [70]. Meanwhile, Gysi *et al.* [68] compared several methods including machine learning from graph embeddings, network diffusion and network proximity. Finding the methods to be complementary to each other, they combined their predictions into a single score and used it to predict repurposable drugs for COVID-19. Then, in order to validate their predictions, they screened 918 drugs against SARS-Cov-2 in the VeroE6 cell line for a large number of drugs and then in human lung cells for the most promising drugs and thus identified several potentially effective drugs which they compared to the list of drugs in clinical trials for COVID-19. In addition to single drug prioritization, drug combinations for treating COVID-19 have also been analyzed [71]. CoVex [102] is a tool developed for exploring the COVID interactome, and it provides various network medicine-related information in an online platform. CoVex utilizes various network approaches including a multi-Steiner tree algorithm to uncover biological pathways related to a given set of viral proteins as well as closeness centrality to find drugs capable of targeting those pathways. Network-based analysis of COVID-19 comorbidities was also considered by many studies in the form of gene set network proximities between diseases [69, 70]. These methods and their highlighted drug repurposing results are summarized in Table 5.

**Table 5:** Drug-repurposing results for COVID-19 from various authors applying network proximity algorithms. Drugs in bold indicate drugs which have FDA emergency use authorization for COVID-19 treatment as listed on https://www.fda.gov/ (accessed 9 February 2022). Curly brackets {} indicate drug combinations

Objective	Network-based approach	Selected drugs	Validation	Ref
Host interactome exploration and drug (target) identification	Various NMD algorithms applied to combined virus-host PPI including: a Steiner tree algorithm, closeness centrality and network proximity (average closest shortest-path-distance between gene sets)	*Starting from the PPI of viral proteins:* Ramipril, Captopril, Perindopril, Enalaprilat, Icatibant, Bradykinin. *Starting from a PPI inducing viral proteins potentially involved in immune response and host genes differentially expressed in SARS-CoV-2 infected cells*: tofacitinib, ruxolitinib, masitinib, erlotinib, sorafenib	Experimental validation data not available. Web application: https://exbio.wzw.tum.de/covex/	[102]
Network medicine drug-repurposing	Ensemble-based approach which is based on three different graph-based approaches: ML combined with graph embeddings, diffusion-based algorithms and proximity-based algorithms.	Auranofin, Azelastine, Vinblastine, Fluvastatin, Methotrexate and Digoxin.	Experimental validation data: *in vitro* - VeroE6 cell line; Huh7 cells. Software: https://github.com/Barabasi-Lab/COVID-19	[68]
Identify drug-disease associations and repurposing drugs	SAveRUNNER aims to quantify the vicinity between the drug targets and the disease-associated proteins in the human interactome via a novel network-based similarity measure that rewards associations between drugs and diseases located in the same network neighborhoods by applying community detection.	SARS & COVID-19: chloroquine, hydroxychloroquine, tocilizumab, heparin COVID-19: {lopinavir, ritonavir, remdesivir, chloroquine, hydroxychloroquine}, dabigatran, adalimumab	Experimental validation data not available. Software: github.com/giuliafiscon/SAveRUNNER	[69]
Identify drug-disease associations in virus-host PPI.	Network proximity (average closest shortest-path-distance between gene sets) in host-virus PPI.	Cefdinir, Toremifene, Irbesartan, Melatonin, Carvedilol.	Experimental validation data not available. Software not available.	[70]
Repurposing single and drug combinations in virus-host PPI.	Network proximity (average closest shortest-path-distance between gene sets) in host-only PPI, complementary exposure for combinations.	Mesalazine, Toremifene, Eplerenone, Paroxetine, Sirolimus, Dactinomycin, irbesartan, mercaptopurine, melatonin, quinacrine, carvedilol, colchicine, camphor, equilin, oxymetholone, emodin {sirolimus, dactinomycin}, {toremifene, emodin}, {mercaptopurine, melatonin}.	Experimental validation data not available. Software not available.	[71]

One key difference with network-based analysis of infectious diseases compared to chronic ones is that the pathogen-host interactome needs to be considered. In this context antiviral drugs can aim to target viral proteins or host proteins [103] while repurposable drugs target host proteins. Regardless, network-based drug discovery can be attempted with or without the pathogen-host interactome. In the present review, [69, 71] used only the host interactome while [68, 70, 104] included interactions between the viral proteins and host proteins identified with affinity-purification mass spectrometry by Gordon *et al.* [105] who also suggested potential drugs for repurposing but considered direct targets rather than the network medicine approach in the wider human interactome.

## Discussion

In this review, we revisit the different data sources and methods that can be used for construction of networks and explore the trends in methods used for analyzing the networks for modeling the effect of drugs and diseases. Precisely, we focus our attention towards databases that curate interaction or association information that can be readily used for construction of networks. We also describe the methods for creation of omics-based networks which are being increasingly used for characterizing diseases for which sufficient experimentally validated interaction data for network construction is not available.

The networks themselves can be utilized in several ways, and the two main categories of methods are those that use network topology to reason about the potential effects of drugs to perform *in silico* screening and those that utilize known drug-disease or drug-target associations to predict unknown associations of the same kind. Conceptually, *in silico* screening is a slightly different problem from drug repurposing. The former can be used to optimize efficacy and safety profiles as desired for repurposing or to pre-screen drugs for *in vitro* testing, while the latter is focused on completing partially observed drug-target and drug-disease association networks under the assumption that known associations are indicative of safe and efficacious drugs for their indicated diseases and that the available information can be used to predict similarly good associations. Thus, drug repurposing methods can use side effects as a source of information for predicting drug-disease associations (e.g. HeTDR) or drug-target associations (e.g. NeoDTI) without explicitly estimating efficacy and safety. On the other hand, efficacy and safety can be estimated with machine learning methods (e.g. PRODeepSyn and Decagon) or by using network topology (e.g. GPSnet and ThETA).

When it comes to drug repurposing in general, network-based approaches include network propagation, matrix factorization, graph embedding or representation learning and the general trend is to use additional sources of information to describe the features of different entities in the networks or the similarities between them. Many recent methods use deep learning approaches, such as autoencoders or graph convolutional networks, to extract features or to directly learn from structured network data in an end-to-end learning process which can be especially powerful as the latent representations will be specifically optimized for the prediction task. The graph convolutional network architecture in particular has many applications in drug repurposing, such as representation learning for graph nodes or whole graphs (such as drug structures). The interpretability of neural networks is often brought up as a potential downside, but attention-based neural network architectures might help researchers gain insight into the biological mechanisms that underlie a successful prediction. The general issues of drug repurposing methods are related to the incompleteness and sparsity of biological and drug-related networks. For drug-disease association prediction especially, the set of known associations is used for training and thus predicting new associations for novel drugs or diseases without known associations can be challenging.

The individual differences between patients are critical component that is often overlooked during modeling the effect of drugs and diseases. The course of formation of a disease can be different in different persons and, accordingly, they might require different treatment strategies. The use of network biology to understand how different biomolecular components are associated to different phenotypes has greatly aided in deciphering the finer differences between patient subgroups and subsequently identify precise therapeutic strategies for the distinct patient/disease categories. Besides this, the use of multi-omics data in characterizing patient-specific networks has also aided towards achieving personalized medicine. Yet, a major limitation of current NDM algorithms for drug discoveries is that they only consider differences in patients based on disease formation and do not consider how different patients even with the same subtype of disease would respond to the drug. Currently, drug response data are limited to very few, specific projects such as GDSC, NCI ALMANAC and LINCS L1000 Connectivity Map, which limit the development of more patient-centered network approaches. However, more large-scale pharmacogenomics and toxicogenomic projects may arise in the future, giving the possibility to implement robust network-based approaches imputing repurposable drugs or drug targets for individual patients or patient subgroups.

## Data availability

No new data were generated or analyzed in support of this research.

**Key Points**

- NDM algorithms have been broadly applied to drug discovery problems. The network medicine paradigm aims to utilize mechanistic relationships to uncover effective drugs that indirectly address disease perturbations.
- Network-based analysis can be used for *in silico* efficacy and safety screening of drugs and drug combinations to lower the cost of drug development.
- The use of heterogeneous networks and node attributes or node similarities can boost drug repositioning accuracy. Matrix factorization and deep learning methods have been adapted to integrate multiple sources of information.
- Networks from patient-specific omics data can aid in precision medicine by identifying disease modules that could be targeted by drugs or by estimating sample-specific drug responses.
- The SARS-CoV-2 pandemic is still an ongoing crisis, and while several treatments for severe COVID-19 have been authorized by the FDA, at the time of writing (28 April 2022) the search for the most effective treatments continues. While most initial clinical trials focused on existing antiviral drugs, network proximity algorithms have been applied to several repurposable drugs acting on a broader set of targets.

## Author Biographies

**Teemu J. Rintala** is an early-stage researcher and doctoral student at the Institute of Biomedicine, University of Eastern Finland. His research interests include bioinformatics and machine learning applications in precision medicine.

**Arindam Ghosh** is an early-stage researcher and doctoral student at the Institute of Biomedicine, University of Eastern Finland. His current research focuses on improving *in silico* assessment of drug efficacy and safety by using network and machine learning-based approaches.

**Vittorio Fortino** is Associate Professor and Head of the Biomedical Informatics research group at the Institute of Biomedicine, University of Eastern Finland. His research interests include bioinformatics, machine learning and network-driven applications for precision medicine.
